# The effect of unpredicted visual feedback on activation in the secondary somatosensory cortex during movement execution

**DOI:** 10.1186/1471-2202-13-138

**Published:** 2012-11-05

**Authors:** Toshiaki Wasaka, Ryusuke Kakigi

**Affiliations:** 1 Department of Integrative Physiology, National Institute for Physiological Sciences, 38 Nishigonaka, Myodaiji, Okazaki, Aichi, 4448585, Japan

**Keywords:** MEG, Sensorimotor integration, Bimanual movement, SII

## Abstract

**Background:**

A mechanism that monitors the congruence between sensory inputs and motor outputs is necessary to control voluntary movement. The representation of limb position is constantly updated on the basis of somatosensory and visual information and efference copy from motor areas. However, the cortical mechanism underlying detection of limb position using somatosensory and visual information has not been elucidated. This study investigated the influence of visual feedback on information processing in somatosensory areas during movement execution using magnetoencephalography. We used an experimental condition in which the visual information was incongruent despite the motor execution and somatosensory feedback being congruent. Subjects performed self-paced bimanual movements of both thumbs, either symmetric or asymmetric, under normal visual and mirrored conditions. The mirror condition provided a visual feedback by showing a reflection of the subject’s right hand in place of the left hand. Therefore, in the Asymmetric task of the Mirror condition, subjects saw symmetric movements despite performing asymmetric movements.

**Results:**

Activation in the primary somatosensory area (SI) revealed inhibition of neural activity and that in the secondary somatosensory area (SII) showed enhancement with voluntary movement. In addition, the SII contralateral to the side of stimulation was significantly enhanced in the Asymmetric task of the Mirror condition, which provided non-veridical visual feedback.

**Conclusions:**

These results suggested that visual information influenced the neuronal activity concerning sensorimotor interaction in the SII during motor execution. The SII contributes to the detection of unpredicted visual feedback of movement execution.

## Background

The information for movement is provided by visual and somatosensory feedback and the integration of cross-modal sensory processing and motor command is critical for motor control. Efficient monitoring of body movement depends on matching the predicted sensory consequences from internal motor commands with actual sensory feedback [1,2]. Normally, visual estimates of limb positions are congruent with somatosensory estimates under normal visual conditions, but a mismatch between watched and felt movements of the hand disrupts motor control. This situation typically produces relatively strong cognitive conflict such as the feeling that movement is not controlled by oneself.

Recently, an increasing number of studies have indicated that cross-modal interaction occurred when neural activity from one sensory modality modulated activity in another [3-5]. Cross-modal links between visual and somatosensory information have shown the critical role of vision in determining limb position and localizing tactile sensations [6-9]. For example, viewing a body part improves tactile perception and facilitates the amplitude of long-latency components of event-related potentials [10-12]. In addition, there is evidence that vision of the body is crucial for localization of tactile stimuli [13,14]. These results indicate that the visual information changes the information processing in somatosensory areas.

Although less attention has been devoted to the effect of observation of movement on information processing in somatosensory areas, some studies have reported neural modulation in the primary somatosensory area (SI) and secondary somatosensory area (SII). Previous studies showed that viewing another person’s gestures modulates the excitability of somatosensory areas [15-18]. These results indicate that the somatosensory areas are involved in the mirroring of actions. In addition, our recent study showed that somatosensory areas have a functional role to detect a mismatch between the intended and actual visual feedback of voluntary movement [19]. However, we could not fully clarify the neural mechanism of cross-modal interaction between visual and somatosensory modalities during movement execution. We must look more carefully into the effect of visual feedback on somatosensory information processing because the mismatch was very different between the expected and actual visual information in previous study. In the Mirror condition, which provided false visual information on movement, the actual visual feedback was a stationary hand and the intended feedback was an image of a moving thumb. To further address this issue, the present study investigated the neural modulation caused by the visual feedback that only differed in the phase of the moving hand.

The aim of the present study was to elucidate sensorimotor mechanisms in detecting incongruence if visual feedback did not match with the predicted one based on somatosensory feedback and motor command. We experimentally manipulated visual feedback using the mirror box technique [20-22]. We created non-intended visual feedback by showing a reflection of the subject’s right hand in place of the left hand during movement execution.

## Methods

### Subjects

Ten healthy volunteers participated in the study (10 males; mean age 35.2 ± 5.3 years, range 28–46 years). None had a history of neurological disorders or took medication before the experiment. All subjects were right-handed as assessed by the Edinburgh Handedness Inventory (average score, 91.1) [23]. Written informed consent was obtained from each participant prior to the study, which was approved by the Ethics Committee at the National Institute for Physiological Sciences, Okazaki, Japan.

### Stimulation

The left median nerve was stimulated on the palm side at the wrist with a saddle-type electrode. The electrode was fixed to the wrist using a belt to prevent it moving during movement execution. The cathode was placed 3 cm proximal to the anode. The electrical stimulus was a constant current square-wave pulse 0.2 ms in duration and the stimulus intensity was 1.1 times the motor threshold of the abductor pollicis brevis muscle, which yielded no pain or unpleasant sensation. The interstimulus interval of electrical stimuli was 3000 ms.

### Experimental conditions

**Figure 1:**
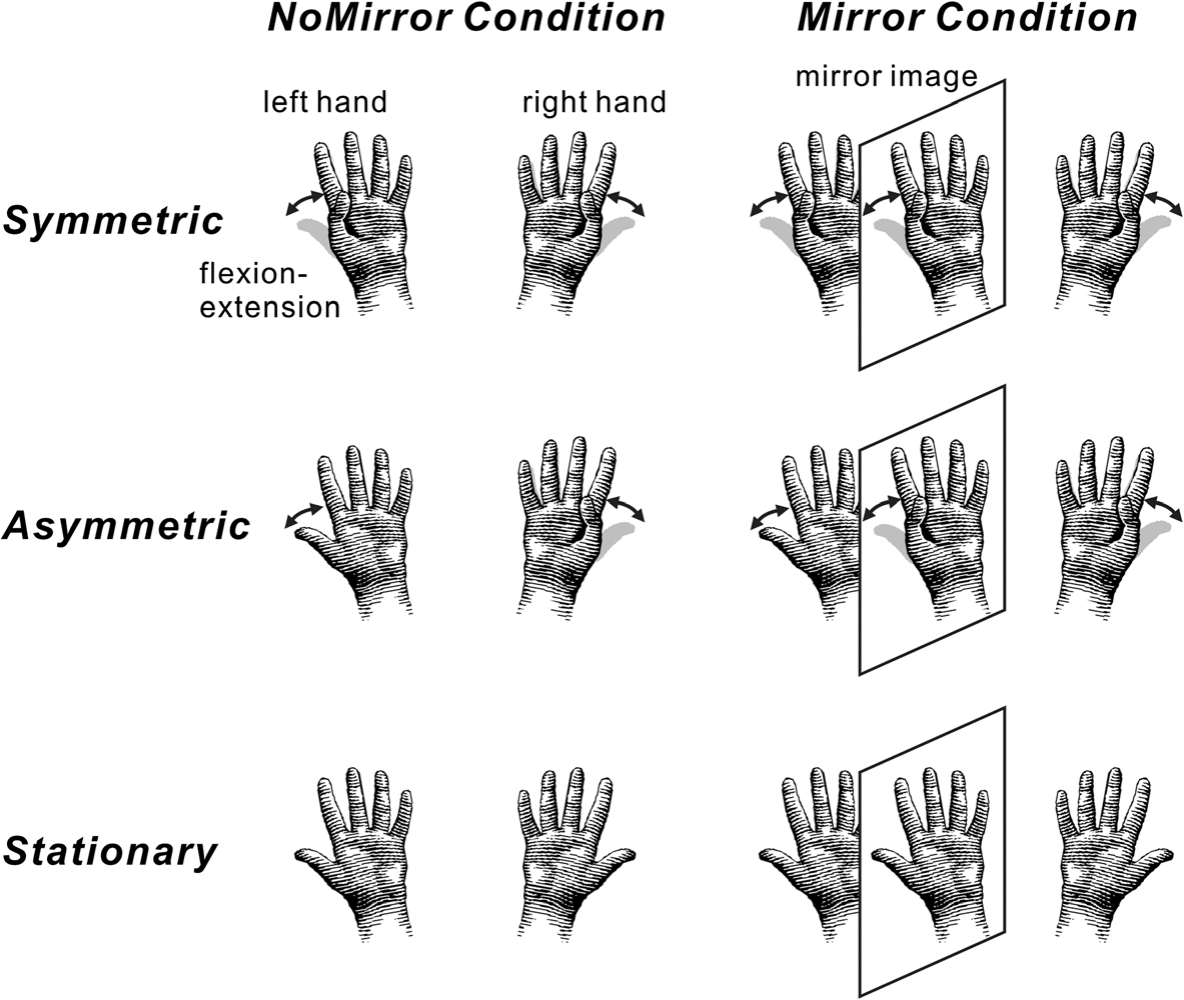
**The experimental paradigm used in this study.** Subjects performed self-paced bimanual movements, either symmetric (Symmetric task) or asymmetric (Asymmetric task), of both thumbs. The other task was that both hands were kept stationary (Stationary task). The three tasks were conducted with normal visual feedback (NoMirror condition) and non-veridical visual feedback (Mirror condition). In the Asymmetric task of the Mirror condition, since the actual visual information on left hand movement was masked by the mirror, subjects saw symmetric movements despite performing asymmetric movements. By contrast, in the Symmetric task, although subjects saw the mirrored movement, the phase of bimanual movement was the same for masked and mirrored hands. Electrical stimulation for the recording of somatosensory responses was delivered to the median nerve at the left wrist.

In this study, we measured brain activities in somatosensory areas while subjects performed two bimanual movement tasks in two conditions (Figure 1). In the Mirror condition, subjects inserted their hands into a mirror box with the forearm supine. The position of the right hand was adjusted so that the mirror image precisely overlapped the view of the masked left hand. A mirror image of the right hand was therefore presented instead of the left hand. In the NoMirror condition, the mirror box was removed. For the magnetoencephalography (MEG) recordings, subjects were instructed to gaze at a small round mark (0.8 cm in diameter) approximately 0.5 m away between the two hands.

In each condition, three tasks were performed. Subjects were instructed to make simple, self-paced repetitive hand movements that involved extending and flexing both thumbs either symmetrically (Symmetric task) or asymmetrically (Asymmetric task). In both tasks, we asked the subjects to perform the sequence of movement (thumb flexion-extension) at a frequency of about 1 Hz. We asked that they did not synchronize their movements with the electrical stimulation. We monitored the thumb movement with a video camera from outside of a shielded room. If the pace of movement was slower or faster than 1 Hz, we gave instructions to the subject. The other task was that both hands were kept stationary (Stationary task). In all tasks, subjects were instructed to pay no attention to the electrical stimulation. In the Mirror condition of the Asymmetric task, since the actual visual information on left hand movement was masked by the mirror, subjects saw symmetric movements despite performing asymmetric movements. In contrast, for the Symmetric task, although subjects saw the mirrored movement, the phase of bimanual movement was the same in masked and mirrored hands. One session of the three tasks comprised 50 stimuli and the recording time of one session was about 3 min. Two sessions were performed, and 100 artifact-free responses were averaged for each condition. The order of tasks in each condition was randomized among the subjects.

### Data acquisition and analysis

The MEG signals were recorded with a helmet-shaped 306-channel detector array (Vectorview, Elekta Neuromag Yo, Helsinki, Finland), which comprised 102 identical triple sensor elements. Each sensor element consisted of two orthogonal planar gradiometers and one magnetometer coupled to a multi-superconducting quantum interference device and thus provided three independent measurements of the magnetic fields. The MEG signals were recorded with a bandpass filter of 0.03-300 Hz and digitized at 1024 Hz. Epochs in which the signal variation was larger than 3000 fT/cm were excluded from the averaging. The period of analysis was from 100 ms before to 250 ms after the onset of the electrical stimulus. The data for 100 ms before the stimulus were used to calculate the baseline.

Prior to the MEG recording, four head position indicator (HPI) coils were placed at specific sites on the scalp. To determine the exact location of the head with respect to the MEG sensors, an electric current was fed to the HPI coils, and the resulting magnetic fields were measured with the MEG sensors. These procedures allowed for alignment of the individual head coordinate system with the MEG coordinate system. The locations of HPI coils with respect to the three anatomical landmarks (nasion and bilateral preauriculas) were also measured using a three-dimensional digitizer to align the coordinate systems of MEG with the T1-weighted magnetic resonance images (MRI) obtained with a 3 tesla MRI system (Allegra; Siemens, Erlangen, Germany). The x-axis was fixed with the preauricular points, the positive direction being to the right. The positive y-axis passed through the nasion and the z-axis thus pointed upward.

**Figure 2:**
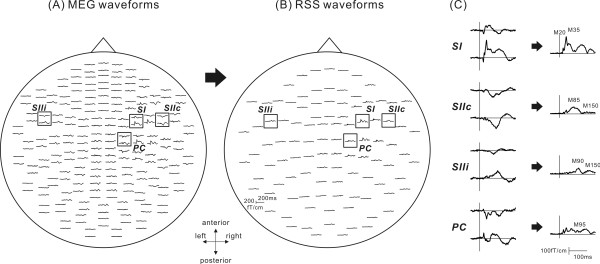
**Method of analysis used in this study.** (**A**) The MEG waveforms viewed from the top of the head following stimulation of the left median nerve recorded from 204 sensors in the Stationary task. Clear deflections were obtained in the central region contralateral to the side stimulated and in the temporal regions in both hemispheres. (**B**) The RSS waveforms from 102 sensors in the stationary task. (**C**) Enlarged MEG and RSS waveforms of the primary somatosensory area (SI), the secondary somatosensory cortex contralateral (SIIc) and ipsilateral to the side stimulated (SIIi), and the parietal cortex (PC). A vertical line indicates the onset of stimulus.

We first calculated vector sums from the longitudinal and latitudinal derivatives of responses recorded on the planar-type gradiometers at each of the 102 sensors’ locations (Figure 2). This was achieved by squaring MEG signals for each of two planar-type gradiometers at a sensor’s location, summing the squared signals together, and then calculating the root of the sum, here we call this the “root sum square” (RSS) [24]. The calculation was carried out for all 102 sensors’ locations. Next, we used the RSS waveforms and a topographic map of RSS amplitude to look for a peak channel showing the greatest amplitude for each prominent response, because the waveforms had several responses with a different spatial distribution of amplitude. Then, the peak amplitude and latency of prominent responses in the RSS waveform were measured at the peak channel.

To identify the equivalent current dipoles (ECDs) in MEG components, sources of measured responses to the electrical stimuli were modeled with the time-varying current dipoles method [25]. Single ECDs of MEG components were estimated. In the period when clear dipolar magnetic field patterns were found, the ECDs that best explained the most dominant signals were determined by a least-squares search, based on 14 to 18 channels around the channel that had been used to measure the peak amplitude of RSS waveforms. The goodness-of-fit (GOF) value of an ECD was calculated to indicate in percentage terms how much the dipole accounted for the measured field variance. Only ECDs which accounted for more than 80% of the GOF in a channel subset were accepted.

For the peak amplitude of the RSS components, to examine whether visual feedback affects the activation of somatosensory areas during bimanual movement, a two-way repeated measures ANOVA was performed with visual condition (Mirror and NoMirror conditions) and movement task (Symmetric, Asymmetric and Stationary tasks) as the factors. To analyze the assumption of sphericity prior to the repeated measures ANOVA, we used Mauchly’s test of sphericity. If a significant test result was obtained and the assumption of sphericity was violated, the Greenhouse-Geisser adjustment was used to correct for the sphericity by altering the degrees of freedom using a correction coefficient epsilon. A post-hoc analysis was conducted using paired t-test. Statistical significance was set at a P value of less than 0.05.

## Results

**Figure 3:**
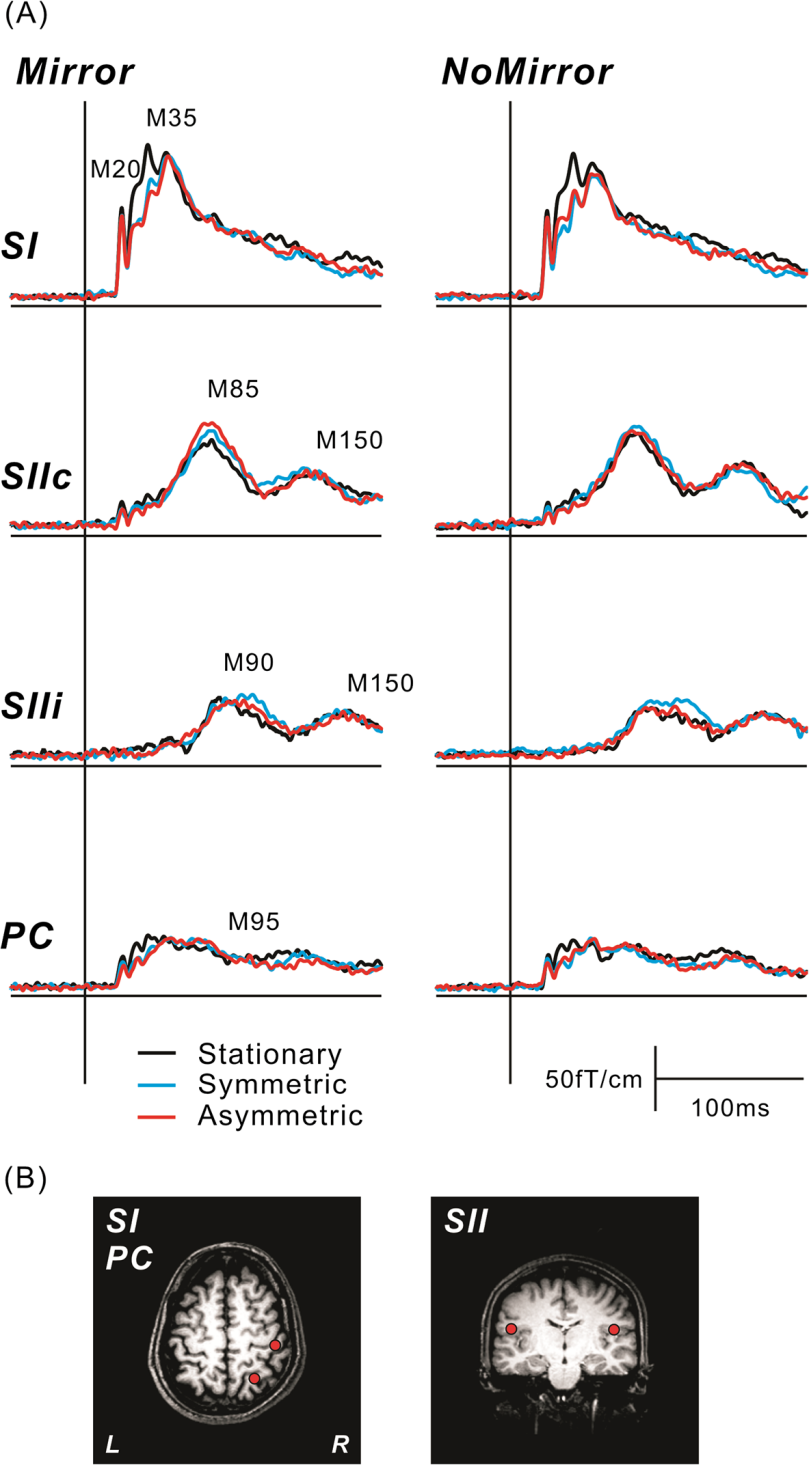
**The grand averaged RSS waveforms and dipole locations.** (**A**) The grand averaged RSS waveforms in each task of the two conditions in the SI, SIIc, SIIi, and PC. A vertical line indicates the onset of stimulus. Prominent responses were observed at around 20 ms (M20) and 35 ms (M35) in the SI, around 85 ms (M85) and 150 ms (M150 SIIc) in the SII contralateral to the side of stimulation, around 90 ms (M90) and 150 ms (M150 SIIi) in the SII ipsilateral to the side stimulated and around 95 ms (M95) in the PC. A reduction in SI components and an enhancement of SII components were identified with movement execution. In addition, the M85 showed significant enhancement in the Asymmetric task of the Mirror condition. (**B**) The location of equivalent current dipoles (ECDs) in each component superimposed on the T1-weighted magnetic resonance images. The ECD for short-latency responses was located in the posterior bank of the central sulcus (SI) in the hemisphere contralateral to the side stimulated, and the long-latency responses (M85 and M90) were identified in the bilateral temporal regions around the upper bank of the sylvian fissure, corresponding to the SII. The source of the parietal activity (M95) was located medial and posterior to the SI hand area in the hemisphere contralateral to the side stimulated.

Figure 2 shows the waveforms of somatosensory evoked fields (SEFs) and RSS in the stationary condition following stimulation of the left median nerve. Five prominent components were observed in RSS waveforms. The earliest deflection of the RSS waveforms was identified in the central region contralateral to the side stimulated at around 20 ms after the stimulation (M20) and the subsequent deflection peaking at around 35 ms (M35). Long-latency components were identified in temporal regions of both hemispheres at around 90 ms (M85 and M90). The ECD for the early responses was located in the posterior bank of the central sulcus corresponding to the SI. The bilateral long-latency responses were estimated to be generated in the upper bank of the sylvian fissure, corresponding to the SII (Figure 3). In addition, around the central region contralateral to the side of stimulation, the sensor was located more posterior than the SI, and a small peak was observed at about 95 ms (M95). The source of the M95 was estimated to be around the parietal cortex (PC) in the posterior wall of the postcentral sulcus.

**Figure 4:**
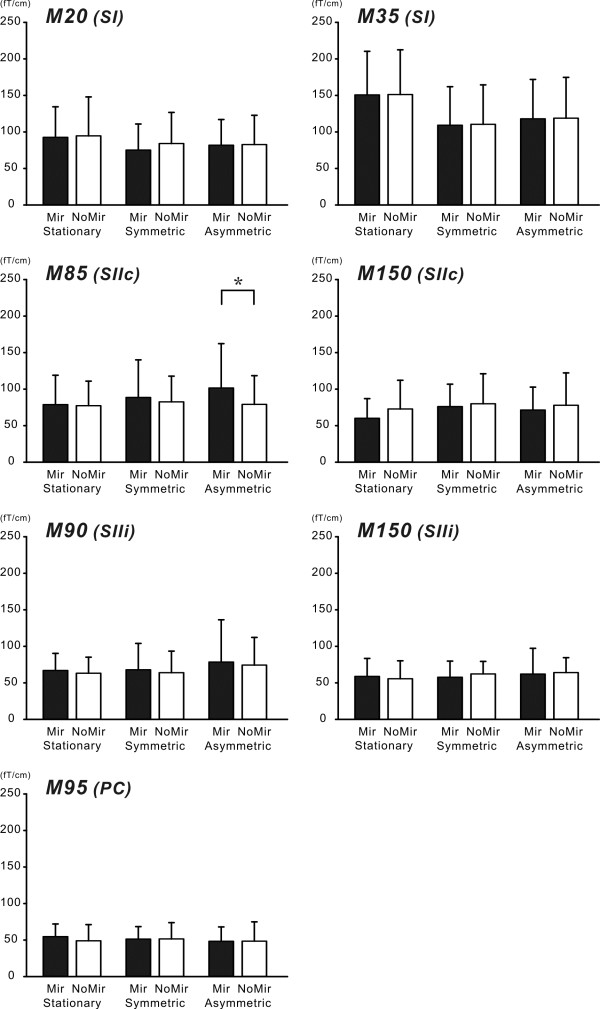
**The modulation of RSS components with voluntary movement in the Mirror and NoMirror conditions.** The y-axis indicates the peak amplitude of RSS component and the error bar means the standard deviation. A two-way repeated measures ANOVA was performed with visual condition (Mirror and NoMirror conditions) and movement task (Symmetric, Asymmetric and Stationary tasks) as the factors. A significant main effect of task was found in the M20 and M35 of the SI. In addition, a significant interaction was found in the M85 of SIIc and M95 of PC. The amplitude of the M85 in the Asymmetric task was significantly larger in the Mirror condition than NoMirror condition. In contrast, no difference was observed in the Symmetric task. *P < 0.05, between two conditions.

Grand-averaged RSS waveforms for each task of the two conditions are shown in Figure 3. To investigate the effect of visual feedback on neural activation in the somatosensory areas during movement execution, we compared the peak amplitude of RSS components using a two-way repeated ANOVA concerning task (Symmetric, Asymmetric and Stationary) and condition (Mirror and NoMirror). Figure 4 shows the peak amplitude of RSS components. A significant main effect of task on the M20 and M35 in the SI was found (M20 F_1,9_ = 7.875, P < 0.05 M35 F_1,9_ = 16.093, P < 0.01), but no significant main effect of condition or interaction. The amplitude of SI components exhibited a reduction during movement execution. In the SII contralateral to the side of stimulation (SIIc) and PC components, although no significant main effect was observed, a significant interaction was found in the M85 (F_2,18_ = 6.425, P < 0.05) and M95 (F_2,18_ = 3.731, P < 0.05). In contrast, no significant change was observed in the M90 and M150 in the SII ipsilateral to the side of stimulation (SIIi) and the M150 in the SIIc. We compared the SIIc component in each task between the Mirror and NoMirror conditions. In the Asymmetric task, the amplitude of the SIIc was significantly higher in the Mirror than NoMirror condition (P < 0.05), while no significant change was observed in the Stationary and Symmetric tasks between the two visual conditions. The non-veridical visual feedback in the Asymmetric task of the Mirror condition affected the activation of the SIIc.

## Discussion

We investigated whether activation in somatosensory areas was affected by the discordance of visual information between intended and executed actions. Subjects received visual feedback about thumb movement that was unintended in the Asymmetric task of the Mirror condition or expected in the Symmetric task of the Mirror condition and NoMirror condition. In the conflict caused by the unintended visual feedback, activation in the SIIc was significantly higher in the Mirror condition than NoMirror condition. The results of this study are in line with our previous findings showing cross-modal interaction between somatosensory and visual modalities in the SII when looking at unintended visual information on a moving hand provided during movement execution [19].

Viewing the body influences the speed of tactile reaction time [26] and improves tactile acuity [27]. Longo et al. [28] reported that the short-latency component of somatosensory evoked potentials (SEPs) generated in the SI was higher on viewing the body than viewing an object. Furthermore, in addition to viewing the body, the observation of moving body parts also modulates the SI activation [15-18], whereas our results showed no changes of SI activity on viewing the unintended visual feedback of body movement. It is well-known that the neural activity in the SI is strongly inhibited during voluntary movement [29-34]. One explanation for the absence of modulation of SI activity with the unexpected visual information in our study may be that the inhibitory effect from motor-related areas canceled out the effect from visual information.

We found that the SIIc response in the Asymmetric task was significantly higher in the Mirror condition than NoMirror condition, while no change was observed in the Symmetric task. The Asymmetric task in both conditions was similar with regard to motor command and somatosensory feedback, and the only difference was the visual feedback. There was a possibility that a very subtle twitch following the stimulation caused the difference in visual feedback between the Mirror and NoMirror conditions. However, since the stimulus intensity was just above the motor threshold, the extension-flexion of the thumb was much larger than the small twitch during movement tasks. It seems reasonable to eliminate the notion that the difference in visual feedback caused by a minor twitch induces the enhancement in the SIIc.

The neural mechanism responsible for modulation of SIIc activities through non-veridical visual feedback is unclear. One possibility is that the incongruent visual information enhances the SIIc activation via an attentional effect. The SII is involved in cross-modal attentional links between the somatosensory and visual modalities [3], and the sight of body parts influences somatosensory event-related potentials with tactile spatial attention [14]. A probable explanation for the modulated neural activations in the SIIc observed in the Mirror condition might be that the visual feedback of unintended phase movement caused by replacement of the subjects’ left hand with a mirror image of the right hand implicitly led to increased attentional demands for the somatosensory information. Another possibility is that the neural mechanism providing the visual information on the body part would be influenced by predictions of visual feedback based on the motor commands. It has been suggested that a copy of the motor signal, known as an efference copy, is created so that sensory signals generated from external stimuli can be distinguished from reafferent signals from body movement [2,35,36]. Corollary discharges are produced only if the motor commands interact with unpredicted sensory inputs and inhibits the neural response to the self-generated sensory signals [37]. More activity in somatosensory areas was found when the unpredicted stimulus was externally delivered [38-40]. There is considerable validity to notion that the prediction of visual feedback modulates the somatosensory areas. In the Asymmetric task in the Mirror condition, subjects faced the surprise of seeing that their hand was not responding as intended, and activation in the SIIc might be modulated by the effect of corollary discharge.

We assume that this modulation in the SIIc is involved in computing the sensory errors by comparing the actual hand’s location to the estimated location for controlling movement. There is another cortical area that is important to a forward model [2]. The cerebellum builds internal models that predict the sensory feedback of motor commands and correct motor commands through internal feedback. The cerebellum also has been proposed to combine information from motor efferent and sensory afferent signals [41]. However, we could not record any cerebellar responses because our whole-head MEG system did not fully cover the cerebellum.

There is evidence that humans are normally not conscious of sensory feedback from movement [42], and are aware that their arms and legs belong to them through somatosensory and visual inputs. This feeling of self-attribution is impaired when the predicted sensory information estimated from motor intention does not match the actual sensory information. In our study, the Asymmetric task of the Mirror condition corresponded to this situation. Some subjects reported feeling that movement in the Asymmetric task of the Mirror condition was not controlled by themselves or the body did not belong to them. Studies in patients and recent neuroimaging results in healthy subjects suggest a prominent role for the posterior parietal cortex [43] and insula [44-47] in the self-awareness of limb actions, the sense of agency. Inui et al. [48] reported simultaneous activation in the contralateral SII and insula peaking at 90 to 160 ms after electrical stimulation. We assumed that the late component peaking at 150 ms (M150) in the SIIc may involve the SII activity of the neighboring insula. However, we could not find significant differences in the insula and PC. Although subjects reported a disturbance of agency, we assumed that it was not enough to induce the difference in these areas. Further study will be needed to clarify the functional role of these areas in sensorimotor integration.

## Conclusions

This study revealed the neural mechanism of somatosensory activation during visual conflict caused by incongruence between the predicted and actual visual feedback in motor control. Our results demonstrated that the SII had cross-modal functions in the somatosensory and visual modalities during motor execution. However, we did not elucidate visuo-tactile interaction in other somatosensory areas such as the posterior parietal cortex and insula. Further research may be useful to elucidate the functional role of somatosensory areas for motor control using somatosensory and visual information.

## Authors’ contributions

TW contributed to planning the study, data collection, analysis, and drafting the paper. RK contributed to planning the study and drafting the paper. Both authors read and approved the final manuscript.

## References

[B1] Shadmehr R Krakauer JW A computational neuroanatomy for motor control Exp Brain Res 2008 185 359 381 10.1007/s00221-008-1280-5 18251019 PMC2553854

[B2] Wolpert DM Miall RC Kawato M Internal models in the cerebellum Trends Cogn Sci 1998 2 338 347 10.1016/S1364-6613(98)01221-2 21227230

[B3] Kida T Inui K Wasaka T Akatsuka K Tanaka E Kakigi R Time-varying cortical activations related to visual-tactile cross-modal links in spatial selective attention J Neurophysiol 2007 97 3585 3596 10.1152/jn.00007.2007 17360823

[B4] Macaluso E Frith CD Driver J Modulation of human visual cortex by crossmodal spatial attention Science 2000 289 1206 1208 10.1126/science.289.5482.1206 10947990

[B5] Schurmann M Kolev V Menzel K Yordanova J Spatial coincidence modulates interaction between visual and somatosensory evoked potentials Neuroreport 2002 13 779 783 10.1097/00001756-200205070-00009 11997686

[B6] vanBeers RJ Sittig AC vanderGon JJD How humans combine simultaneous proprioceptive and visual position information Exp Brain Res 1996 111 253 261 8891655 10.1007/BF00227302

[B7] Botvinick M Cohen J Rubber hands ‘feel’ touch that eyes see Nature 1998 391 756 10.1038/35784 9486643

[B8] Graziano MSA Where is my arm? The relative role of vision and proprioception in the neuronal representation of limb position P Natl Acad Sci USA 1999 96 10418 10421 10.1073/pnas.96.18.10418 PMC17903 10468623

[B9] MonWilliams M Wann JP Jenkinson M Rushton K Synaesthesia in the normal limb P Roy Soc B-Biol Sci 1997 264 1007 1010 10.1098/rspb.1997.0139 PMC1688538 9263468

[B10] Cardini F Longo MR Haggard P Vision of the body modulates somatosensory intracortical inhibition Cereb Cortex 2011 21 2014 2022 10.1093/cercor/bhq267 21285259

[B11] Kennett S Spence C Driver J Visuo-tactile links in covert exogenous spatial attention remap across changes in unseen hand posture Percept Psychophys 2002 64 1083 1094 10.3758/BF03194758 12489663

[B12] Taylor-Clarke M Kennett S Haggard P Vision modulates somatosensory cortical processing Curr Biol 2002 12 233 236 10.1016/S0960-9822(01)00681-9 11839277

[B13] Eimer M Forster B Fieger A Harbich S Effects of hand posture on preparatory control processes and sensory modulations in tactile-spatial attention Clin Neurophysiol 2004 115 596 608 10.1016/j.clinph.2003.10.015 15036056

[B14] Sambo CF Gillmeister H Forster B Viewing the body modulates neural mechanisms underlying sustained spatial attention in touch Eur J Neurosci 2009 30 143 150 10.1111/j.1460-9568.2009.06791.x 19519638

[B15] Avikainen S Forss N Hari R Modulated activation of the human SI and SII cortices during observation of hand actions NeuroImage 2002 15 640 646 10.1006/nimg.2001.1029 11848707

[B16] Mottonen R Jarvelainen J Sams M Hari R Viewing speech modulates activity in the left SI mouth cortex NeuroImage 2005 24 731 737 10.1016/j.neuroimage.2004.10.011 15652308

[B17] Pihko E Nangini C Jousmaki V Hari R Observing touch activates human primary somatosensory cortex Eur J Neurosci 2010 31 1836 1843 10.1111/j.1460-9568.2010.07192.x 20584188

[B18] Rossi S Tecchio F Pasqualetti P Ulivelli M Pizzella V Romani GL Passero S Battistini N Rossini PM Somatosensory processing during movement observation in humans Clin Neurophysiol 2002 113 16 24 10.1016/S1388-2457(01)00725-8 11801420

[B19] Wasaka T Kakigi R Conflict caused by visual feedback modulates activation in somatosensory areas during movement execution NeuroImage 2012 59 1501 1507 10.1016/j.neuroimage.2011.08.024 21889595

[B20] Altschuler EL Wisdom SB Stone L Foster C Galasko D Llewellyn DM Ramachandran VS Rehabilitation of hemiparesis after stroke with a mirror Lancet 1999 353 2035 2036 10.1016/S0140-6736(99)00920-4 10376620

[B21] Ramachandran VS Rogers-Ramachandran D Synaesthesia in phantom limbs induced with mirrors Proc Biol Sci 1996 263 377 386 10.1098/rspb.1996.0058 8637922

[B22] Ramachandran VS Rogers-Ramachandran D Cobb S Touching the phantom limb Nature 1995 377 489 490 10.1038/377489a0 7566144

[B23] Oldfield RC The assessment and analysis of handedness: the Edinburgh inventory Neuropsychologia 1971 9 97 113 10.1016/0028-3932(71)90067-4 5146491

[B24] Kida T Wasaka T Inui K Akatsuka K Nakata H Kakigi R Centrifugal regulation of human cortical responses to a task-relevant somatosensory signal triggering voluntary movement NeuroImage 2006 32 1355 1364 10.1016/j.neuroimage.2006.05.015 16806987

[B25] Hamalainen M Hari R Ilmoniemi R Kunuutila J Lounasmaa OV Magnetoencephalography-Theory, instrumentation, and application to noninvasive studies of the working human brain Rev Mod Phys 1993 65 413 497 10.1103/RevModPhys.65.413

[B26] Tipper SP Lloyd D Shorland B Dancer C Howard LA McGlone F Vision influences tactile perception without proprioceptive orienting Neuroreport 1998 9 1741 1744 10.1097/00001756-199806010-00013 9665593

[B27] Kennett S Taylor-Clarke M Haggard P Noninformative vision improves the spatial resolution of touch in humans Curr Biol 2001 11 1188 1191 10.1016/S0960-9822(01)00327-X 11516950

[B28] Longo MR Pernigo S Haggard P Vision of the body modulates processing in primary somatosensory cortex Neurosci Lett 2011 489 159 163 10.1016/j.neulet.2010.12.007 21147197

[B29] Kakigi R Koyama S Hoshiyama M Watanabe S Shimojo M Kitamura Y Gating of somatosensory evoked responses during active finger movements magnetoencephalographic studies J Neurol Sci 1995 128 195 204 10.1016/0022-510X(94)00230-L 7738596

[B30] Kakigi R Shimojo M Hoshiyama M Koyama S Watanabe S Naka D Suzuki H Nakamura A Effects of movement and movement imagery on somatosensory evoked magnetic fields following posterior tibial nerve stimulation Brain Res Cogn Brain Res 1997 5 241 253 10.1016/S0926-6410(97)00002-5 9088560

[B31] Rushton DN Rothwell JC Craggs MD Gating of somatosensory evoked potentials during different kinds of movement in man Brain 1981 104 465 491 10.1093/brain/104.3.465 7272711

[B32] Wasaka T Hoshiyama M Nakata H Nishihira Y Kakigi R Gating of somatosensory evoked magnetic fields during the preparatory period of self-initiated finger movement NeuroImage 2003 20 1830 1838 10.1016/S1053-8119(03)00442-7 14642492

[B33] Wasaka T Kida T Nakata H Akatsuka K Kakigi R Characteristics of sensori-motor interaction in the primary and secondary somatosensory cortices in humans: a magnetoencephalography study Neuroscience 2007 149 446 456 10.1016/j.neuroscience.2007.07.040 17869442

[B34] Wasaka T Nakata H Akatsuka K Kida T Inui K Kakigi R Differential modulation in human primary and secondary somatosensory cortices during the preparatory period of self-initiated finger movement Eur J Neurosci 2005 22 1239 1247 10.1111/j.1460-9568.2005.04289.x 16176367

[B35] Desmurget M Grafton S Forward modeling allows feedback control for fast reaching movements Trends Cogn Sci 2000 4 423 431 10.1016/S1364-6613(00)01537-0 11058820

[B36] von Holst E Mittelstaedt H Das Reafferenzprinzip: Wechselwirkungen Zwischen Zentralnervensystem und Peripherie Natursissenschaften 1950 37 464 476 10.1007/BF00622503

[B37] Sperry RW Neural basis of the spontaneous optokinetic response produced by visual inversion J Comp Physiol Psychol 1950 43 482 489 14794830 10.1037/h0055479

[B38] Blakemore SJ Goodbody SJ Wolpert DM Predicting the consequences of our own actions: the role of sensorimotor context estimation J Neurosci 1998 18 7511 7518 9736669 10.1523/JNEUROSCI.18-18-07511.1998 PMC6793221

[B39] Blakemore SJ Wolpert DM Frith CD Central cancellation of self-produced tickle sensation Nat Neurosci 1998 1 635 640 10.1038/2870 10196573

[B40] Hesse MD Nishitani N Fink GR Jousmaki V Hari R Attenuation of somatosensory responses to self-produced tactile stimulation Cereb Cortex 2010 20 425 432 10.1093/cercor/bhp110 19505992

[B41] Miall RC Christensen LO Cain O Stanley J Disruption of state estimation in the human lateral cerebellum PLoS Biol 2007 5 e316 10.1371/journal.pbio.0050316 18044990 PMC2229864

[B42] Fourneret P Jeannerod M Limited conscious monitoring of motor performance in normal subjects Neuropsychologia 1998 36 1133 1140 10.1016/S0028-3932(98)00006-2 9842759

[B43] Farrer C Frey SH Van Horn JD Tunik E Turk D Inati S Grafton ST The angular gyrus computes action awareness representations Cereb Cortex 2008 18 254 261 17490989 10.1093/cercor/bhm050

[B44] Farrer C Franck N Georgieff N Frith CD Decety J Jeannerod M Modulating the experience of agency: a positron emission tomography study NeuroImage 2003 18 324 333 10.1016/S1053-8119(02)00041-1 12595186

[B45] Farrer C Frith CD Experiencing oneself vs another person as being the cause of an action: the neural correlates of the experience of agency NeuroImage 2002 15 596 603 10.1006/nimg.2001.1009 11848702

[B46] Karnath HO Baier B Right insula for our sense of limb ownership and self-awareness of actions Brain Struct Funct 2010 214 411 417 10.1007/s00429-010-0250-4 20512380

[B47] Karnath HO Baier B Nagele T Awareness of the functioning of one’s own limbs mediated by the insular cortex? J Neurosci 2005 25 7134 7138 10.1523/JNEUROSCI.1590-05.2005 16079395 PMC6725240

[B48] Inui K Wang X Qiu Y Nguyen BT Ojima S Tamura Y Nakata H Wasaka T Tran TD Kakigi R Pain processing within the primary somatosensory cortex in humans Eur J Neurosci 2003 18 2859 2866 10.1111/j.1460-9568.2003.02995.x 14656335

